# In-vitro characterization of a cochlear implant system for recording of evoked compound action potentials

**DOI:** 10.1186/1475-925X-11-22

**Published:** 2012-04-25

**Authors:** Christian Neustetter, Matthias Zangerl, Philipp Spitzer, Clemens Zierhofer

**Affiliations:** 1 C. Doppler Laboratory for Active Implantable Systems, Institute of Ion Physics and Applied Physics, University of Innsbruck, Technikerstr. 25, Innsbruck A-6020, Austria; 2 Research and Development, MED-EL GmbH, Fürstenweg 77a, Innsbruck A-6020, Austria

**Keywords:** Cochlear implant, EAP recording system, linearity, resolution, MED-EL PULSARCI^100^, Sigma-delta modulation, Adaptive sigma-delta modulation, Noise shaping, ECAP

## Abstract

**Background:**

Modern cochlear implants have integrated recording systems for measuring electrically evoked compound action potentials of the auditory nerve. The characterization of such recording systems is important for establishing a reliable basis for the interpretation of signals acquired in vivo. In this study we investigated the characteristics of the recording system integrated into the MED-EL PULSARCI^100 ^cochlear implant, especially its linearity and resolution, in order to develop a mathematical model describing the recording system.

**Methods:**

In-vitro setup: The cochlear implant, including all attached electrodes, was fixed in a tank of physiologic saline solution. Sinusoidal signals of the same frequency but with different amplitudes were delivered via a signal generator for measuring and recording on a single electrode.

Computer simulations: A basic mathematical model including the main elements of the recording system, i.e. amplification and digitalization stage, was developed. For this, digital output for sinusoidal input signals of different amplitudes were calculated using in-vitro recordings as reference.

**Results:**

Using an averaging of 100 measurements the recording system behaved linearly down to approximately -60 dB of the input signal range. Using the same method, a system resolution of 10 μV was determined for sinusoidal signals. The simulation results were in very good agreement with the results obtained from in-vitro experiments.

**Conclusions:**

The recording system implemented in the MED-EL PULSARCI^100 ^cochlear implant for measuring the evoked compound action potential of the auditory nerve operates reliably. The developed mathematical model provides a good approximation of the recording system.

## Background

Cochlear implants (CIs) are prostheses that aim to facilitate auditory perception and speech understanding in patients suffering from profound hearing loss. In normal hearing persons, hair cells inside the cochlea transform acoustic signals into complex patterns of neural signals. These are then transported along the auditory nerve to the brain where they are perceived as sound. In a typical CI patient the hair cells are damaged or absent. CIs make up for this loss by delivering electrical pulses to electrodes located inside the cochlea, which in turn stimulate auditory nerve cells to elicit hearing sensations [1,2]. Many studies have demonstrated that cochlear implantation improves the daily life of patients [3-5], and in particular allows young children with congenital deafness to experience almost normal hearing and speech development [6,7]. Other studies have shown that patients with single sided deafness and suffering from tinnitus can also benefit from cochlear implantation [8-10].

**Figure 1:**
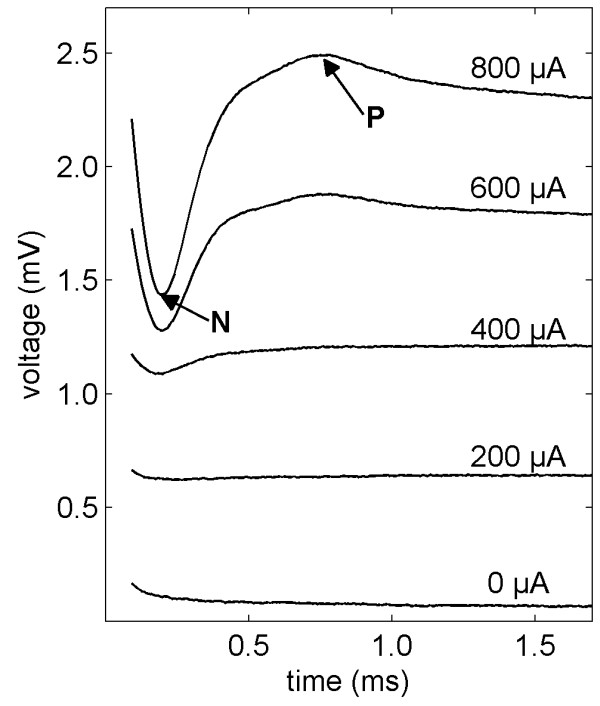
**EAP signals obtained from a human subject**. EAP signals recorded with different stimulation currents from a human subject implanted with the MED-EL PULSARCI^100 ^cochlear implant [17]. The EAP is detectable for stimulation currents above a certain threshold level, and the EAP amplitude increases with higher stimulation. The stimulation current values are indicated next to the corresponding EAP recordings. Signals are plotted at different offsets to clearly distinguish them. The zero-point of the time axis corresponds to the starting point of the recording.

Good CI performance requires the relevant neural structures to be functioning, therefore there is a demand for objective measures to be able to diagnose the state of these structures. This need is met by modern CIs, as they incorporate recording systems for measuring the responses of the neurons. In electrical stimulation the action potentials of different neurons are elicited simultaneously by a single current pulse. The electrical signals resulting from the combination of these action potentials are known as electrically evoked compound action potentials (EAPs) [11,12]. EAP signals measured in a CI user are shown in Figure 1. EAPs typically appear within a millisecond after the onset of a stimulation pulse and show characteristic waveforms with amplitudes (i.e. the voltage difference between minimum N and maximum P) up to approximately one millivolt [13]. EAP measurements are usually carried out for determining the amplitude growth function and the recovery function [14]. EAPs are also used to assist in the implant fitting procedure (i.e. determination of the patient-dependent current limits necessary for normal operation of the implant) [15,16], which is especially desirable for potentially non-cooperative patients, such as cognitively impaired individuals or young children [12].

The demands on such EAP recording systems are very high: Signal waveforms with small amplitudes have to be acquired with high temporal resolution and in the presence of interfering signals like stimulation artifacts, which occur as a result of stimulation current and amount to voltages of several 100 mV at the recording electrode. Therefore, recording systems must be designed carefully to prevent an overload on system components due to these large voltages. The characterization of such recording systems is important for establishing a reliable basis for the interpretation of signals acquired in vivo. The EAP recording system of the MED-EL i100-core family is based on sigma-delta modulation, which is a standard technique for analog-to-digital conversion [18]. The Sigma-Delta Modulator (SDM) implemented in the tested implant can be operated in standard or adaptive mode [19]. This recording system is implemented in the MED-EL cochlear implants of the type PULSARCI^100^, SONATATI^100 ^and CONCERTO [20]. The aim of this study was to characterize the EAP recording system implemented in the MED-EL PULSARCI^100 ^cochlear implant with regard to system linearity and resolution. In addition, a mathematical model was created to appropriately describe the tested recording system.

## Methods

Testing was performed by means of in-vitro experiments which comprised of a simple setup replicating a nearly realistic EAP recording situation. In this study we used sinusoidal signals which are a standard signal type for system characterization purposes.

In order to develop a mathematical description of the tested EAP recording system, a model including the relevant system parts was defined. The results of the corresponding simulations were used as a reference for the interpretation of the results obtained from the in-vitro recordings.

### Measurement setup

**Figure 2:**
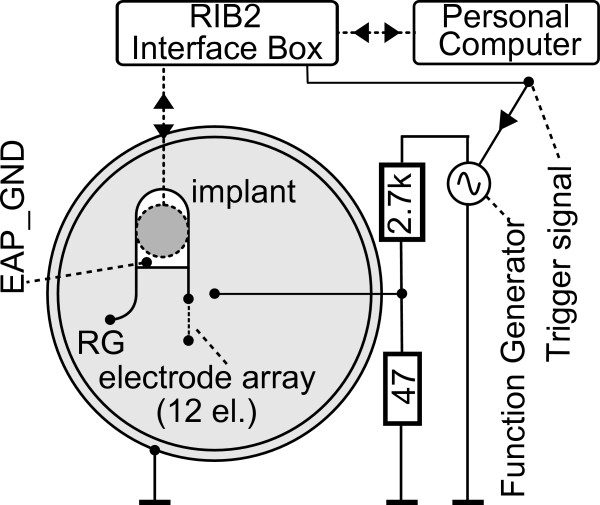
**Schema of the experimental setup**. A PULSARCI^100 ^cochlear implant and an electrode array was placed in a petri dish filled with physiologic saline solution. The external signal was provided by a signal generator. Communication between the implant and the PC is accomplished by the RIB2.

A schematic drawing of the experimental setup is provided in Figure 2. For the in-vitro experiments a MED-EL PULSARCI^100 ^cochlear implant [21] (designated as "implant" hereafter) was placed in a Petri dish filled with a physiologic saline solution. The Petri dish was equipped with a copper cylinder electrode on the inner boundary surface and a copper stick electrode in the centre for presenting an external signal which served as an artificial EAP signal. The external signal was delivered by a signal generator (Rohde&Schwarz, model AFG), which had to be coupled to the petri dish via a voltage divider because of its output range. The generator output was measured to determine imperfections in the external signal source like the offset and higher harmonic components of the main signal frequency. A data acquisition device (National Instruments PCI-6024E) with a sampling rate of 200 kHz and a resolution of 12 bits was used for this purpose. The electrode configuration within the petri dish caused the signal amplitude to be dependent on the radial position of the recording electrodes of the implant. Therefore, the implant and the electrodes were fixed with a plastic holding device and plastic eyelets which were glued on to the bottom of the petri dish. Similar to the setup described by Bahmer [22], the overall procedure and the communication to a host PC were controlled within Matlab (The MathWork, Inc.) via the "Research Interface Box 2" (RIB2), which was developed at the Department of Ion Physics and Applied Physics, University of Innsbruck. The RIB2 consists of software and hardware components for communication with MED-EL implants.

Additional testing of implant hardware was performed with a pin-board version of the electronic chip implemented in the tested implant. This provided several input and output channels of the Application Specific Integrated Circuit (ASIC) of the implant.

### Implant

**Figure 3:**
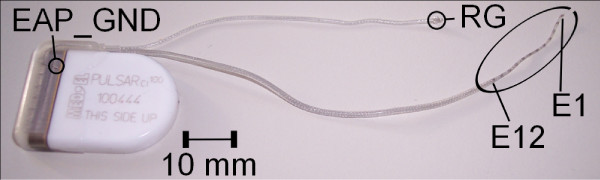
**The PULSARCI^100 ^cochlear implant with the standard electrode array**. The most apical electrode pair (*E1*) and the most basal electrode pair (*E12*) of the intra-cochlear electrode array are labelled. The reference electrodes for stimulation (RG) and EAP measurements (EAP_GND) are also shown. The electronic parts are encased in a ceramic housing.

Figure 3 shows the tested implant with the intra-cochlear and reference electrodes. The electronic components (application specific integrated circuit, current sources, receiver coil) are protected within the flat ceramic housing. Measurements were performed with an implant equipped with the MED-EL Standard Electrode Array [14]. This comprises of twelve pairs of electrodes arranged in a linear array, with an electrode spacing of 2.4 mm. In our experiments we used the same numbering scheme of the intra-cochlear electrodes as in the clinical system, i.e. E1 identifies the most apical electrodes while E12 identifies the most basal ones. The reference electrode for monopolar stimulation is called the Remote Ground (RG), and in the tested implant is designed with a clover-leaf shape.

Other than the electronic components for stimulation, the implant also includes electronic components for measuring purposes, such as for the recording of EAPs. For EAP measurements the recording electrode needs to be different from the stimulation electrode as large transients due to residing charge at the stimulation electrode distort the measurement. The reference electrode for EAP recordings (EAP_GND) is different form RG and is located in the implant housing.

**Figure 4:**
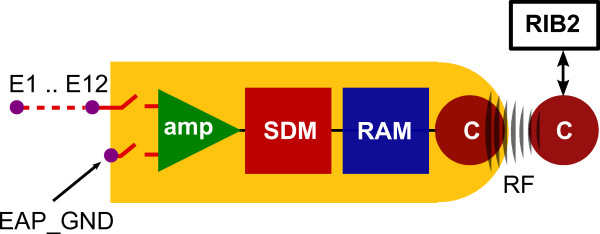
**The main components of the EAP recording system**. Illustration of the main components of the EAP recording system integrated in the MED-EL PULSARCI^100^cochlear implant (*E1...E12: *measuring electrodes, *EAP_GND: *reference electrode for EAP recordings, *amp: *amplifier, *SDM: *sigma-delta modulator, *RAM: *2048 bit storage, *C: *transmitter and receiver coils). The recording window has a maximum duration of approximately 1.7 ms which starts after an adjustable interval after the preceding stimulation pulse. The SDM can be programmed in standard or adaptive mode. The "Research Interface Box 2" (RIB2) is used as an interface with a computer.

Figure 4 illustrates the main parts and the signal processing chain of the EAP recording system. In EAP experiments stimulation generally causes voltages of several 100 mV at the recording electrodes depending on the stimulation amplitude and electrode impedance. To prevent the amplifier from being overloaded due to this large voltage, the recording electrodes are disconnected at the beginning of stimulation and then switched on for recording after the stimulation pulse is finished. The signal appearing at the intra-cochlear electrode in reference to the EAP_GND is amplified by an instrumentation amplifier that has a fixed gain of 40 dB and a bandwidth of 12 kHz. In this band the amplifier noise amounts to 6 μV/Hz^1/2^. In order to reduce the offset of the instrumentation amplifier, prior to recording the system measures the offset, which is subtracted automatically from the input signal (auto-offset calibration). The amplified analog signal is digitzed into a two-value binary sequence at a sampling rate of 1.2 MHz by means of an SDM with a one-bit quantizer. There are two SDM modes available. Other than the standard mode which is implemented as a first-order SDM, the system can also be programmed in adaptive mode. This mode uses an adaptive SDM [19], which is a first-order SDM with a "predictor signal" feedback. Compared to the standard mode, the adaptive mode features 12 dB lower quantization noise but a moderate slew rate limitation [19]. In both modes the quantization noise is frequency dependent and increases with a rate of 20 dB/decade towards higher frequencies. The input signal range of the EAP recording system is ± 5.5 mV in both SDM modes. The two-value binary sequence transmitted from the SDM is stored on an on-chip memory that has a size of 2048 bits. This results in a maximum recording duration of 1.7 ms in both SDM modes. The stored binary sequence can be read out anytime by launching the read back command. For reading back the memory requires a duration of 6.9 ms. The electronic components for recording signals operate independently from the electronic components for stimulation. Thus, the combination of the stimulation and recording systems implemented in this implant offers a huge range of flexibility for EAP experiments, e.g. for implementing different artifact reduction strategies [22].

### Procedure

**Figure 5:**
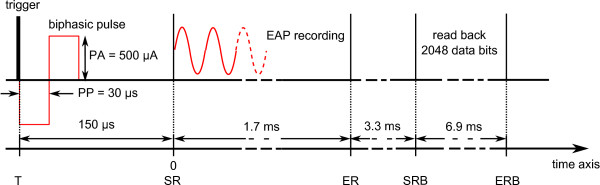
**Timing parameters of the EAP recordings used in the experiments**. The signal is recorded 150 μs after the beginning of the biphasic cathodic-first stimulation pulse that has an amplitude of 500 μA (pulse phase duration: PP = 30 μs, inter phase gap: IPG = 2.1 μs). A period of 1.7 ms is recorded, and read back of the data takes 6.9 ms.

Details of the timing parameters used for the EAP recordings are illustrated in Figure 5. For every measurement, the implant generated a single biphasic stimulation pulse at electrode E1. In this study, a cathodic-first biphasic pulse with a phase duration of 30 μs and an inter-phase gap of 2.1 μs was used. A stimulation amplitude of 500 μA was used, which is within the current range for which EAPs were recorded by Brill [14]. A trigger generated by the RIB2 at the beginning of the stimulation pulse provoked the signal generator to deliver a sinusoidal burst at a frequency of 4.3 kHz. This signal frequency was chosen because; it fell within the bandwidth of the instrumentation amplifier; it was related to the components of a typical EAP signal; and was a high enough frequency to cover a worst case situation, as quantization noise increases towards higher frequencies. The duration of this burst was approximately 2.8 ms, which is slightly longer than the duration of the recording. The amplitude of the sinusoidal burst was first adjusted for the recorded signal to exploit the input signal range. Then, in order to assess the overall performance of the EAP recording system the amplitude was reduced in steps of 6 dB.

The recording was started 150 μs after the beginning of the stimulation pulse, and E2 was used as the measuring electrode. After each recording and the transmitting of the read-back command, the binary sequence was read back via the RIB2 interface and stored on a computer. A series of 100 recordings were performed for each signal amplitude and SDM mode.

### Data analysis

For further analysis of the recordings the corresponding SDM outputs were used. It should be noted that the SDM outputs are different in standard and adaptive mode. In standard mode the SDM output is simply the two value binary sequence, while in adaptive mode the SDM output is a multi-bit signal which is reconstructed from the two-value binary sequence [19]. The measurements taken with each mode were analysed for single recordings and for calculating averages. For the latter we used the mean SDM output, which was calculated by averaging 100 SDM outputs in each series of measurements.

### Waveforms and spectra

The waveforms of the in-vitro recordings were obtained by low-pass filtering the SDM outputs. The applied FIR-filter features a cut-off frequency of approximately 8 kHz with a slope steeping approximately -120 dB/octave, and an impulse response with a duration of 0.3 ms. The waveforms of the generator output were transmitted in the same way as they were delivered by the data acquisition device, without extra filtering or averaging being done.

The spectra of the in-vitro recordings were obtained by discrete Fourier transformation of the SDM output (Matlab FFT, 2048 data points). In order to reduce a leakage effect due to the limited duration of the recording, the window function *g^0^_3.5 _= (cos(πt))^5 ^rect(t) *as suggested by Zierhofer [23] was used for calculating the spectra. This window function features a side lope ripple decay of 120 dB/decade.

The spectra of the generator were calculated by discrete Fourier transformation of the generator output (Matlab FFT, 340 data points). Therefore, only the measured waveforms occurring within a recording period of 1.7 ms and in the same window function as mentioned above were used.

The remaining figures in this paper depict the magnitude spectra which is simply referred to as the "spectra".

### Linearity and resolution

In order to determine the linearity of the recording system, we used the results obtained from the spectra. The amplitude of the main signal components at 4.3 kHz were plotted against the amplitude of the corresponding generator signals. Thus, for an ideal system behaving in a linear way the data points of such a diagram are expected to follow a straight line.

The resolution of the recording system was determined based on the reproducibility of the waveforms obtained in-vitro. As a measure of reproducibility, we calculated the mean variance of all possible pairs of waveforms within the same measurement series i.e. all waveforms obtained with the same amplitude of the generator signal. The variance in each pair of waveforms was calculated as the two-fold standard deviation of the difference signal, which was obtained by subtracting the waveforms from each other. For this calculation only the section of the waveforms that were not contaminated by filter onset-offset effects were used. Using the described procedure we estimated the resolution of the system for waveforms obtained from single recordings and those obtained from the average of 100 recordings.

### Simulations

**Figure 6:**
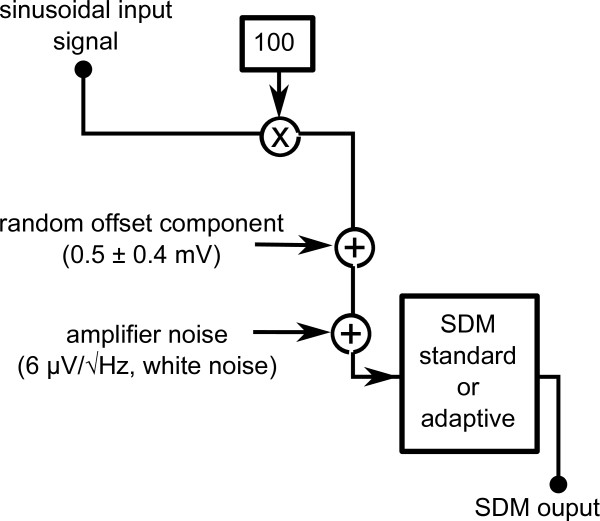
**Mathematical Model**. The block diagram illustrates the main parts of the mathematical model used for the computer simulations of the EAP recording system: Sinusoidal input signals are processed by an amplification (40 dB gain) and digitalization stage (first-order or adaptive software SDM). The model also takes random offset components and amplifier noise into account. The corresponding waveforms and spectra are obtained from the SDM outputs.

The main components of the mathematical model of the EAP recording system are described in the block diagram depicted in Figure 6. In this model a linear amplifier with a gain of 40 dB and amplifier noise of 6 μV/Hz^1/2^, and the SDM were used. Considering the results obtained from the in-vitro recordings, random offset components in the range of 0.5 ± 0.4 mV (see results section) were added to the model. The signal was then processed by the SDM which delivered the output of the two-value binary sequence for standard mode and the multi-bit signal for adaptive mode. Following on from this model, SDM outputs were calculated for sinusoidal signal bursts of different amplitudes. The largest amplitude of these input signals was matched to the largest amplitude measured in-vitro, and the subsequent input signals were determined by gradually reducing the amplitude in 6 dB steps. For simulating a measurement series of 100 recordings with the same burst amplitude, each signal was processed 100 times but with different amplifier noise and a different value of the random offset component. The waveforms and spectra were obtained from the SDM outputs using the same method, and the same filter and window function as used for in-vitro recordings. The software used for calculating the simulations and signal analysis was implemented in Matlab.

## Results and discussion

### Generator output

**Figure 7:**
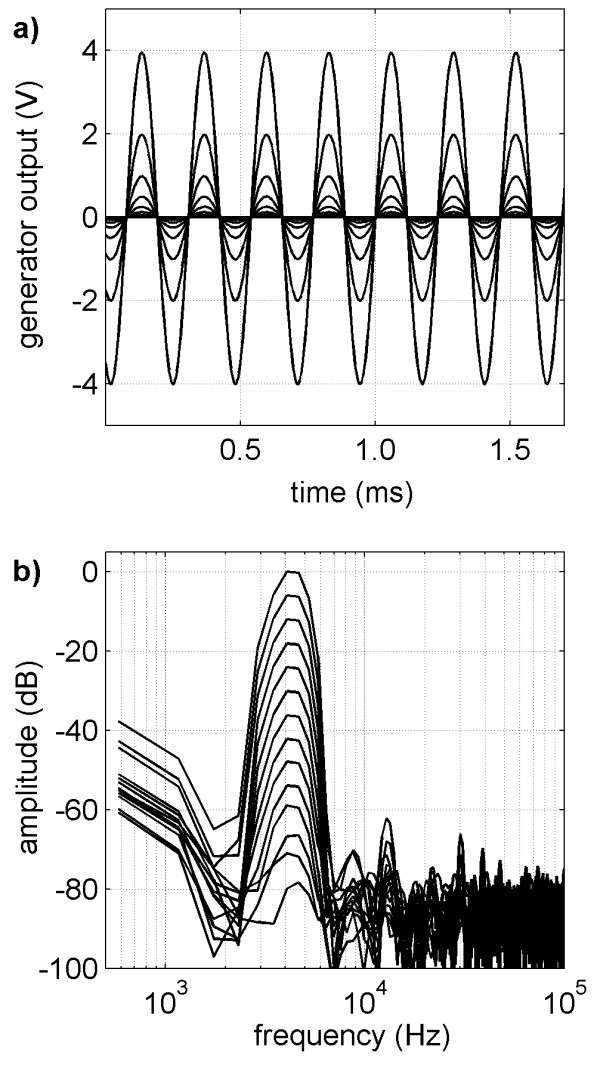
**Generator signals**. Generator signals: a) Waveforms of the sinusoidal signal burst provided at the generator output during the recording window of the implant. The generator output signals were sampled using an analog measuring device (National Instruments PCI-6024E) with a rate of 200 kHz and a resolution of 12 bits. The largest signal amplitude generated was 4 V. b) Corresponding spectra of the generator outputs for different amplitudes with the common main signal frequency of 4.3 kHz.

The waveforms and spectra of the generator output signals at different amplitudes are shown in Figure 7. The main frequency component at 4.3 kHz can be clearly distinguished from the offset and higher harmonic components resulting from imperfections in the generator. The observed offset component of the generator was not relevant as analysis was based on the main signal component of the spectra and the alternating part of the waveforms. The higher harmonic components of the generator signals with the largest amplitude were observed.

### Single recordings with standard and adaptive mode

**Figure 8:**
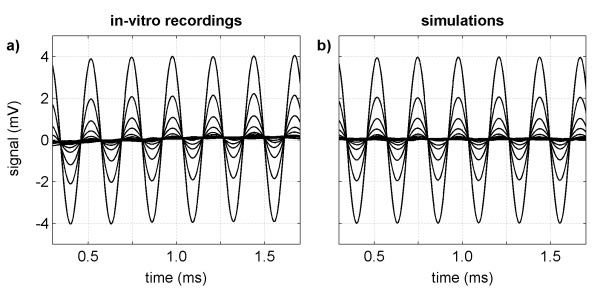
**Waveforms obtained from single SDM outputs**. Waveforms obtained from single in-vitro recordings in adaptive mode (a) and the corresponding simulations (b) of different input signal amplitudes.

Pilot experiments using different electrodes of the intra-cochlear array for measuring EAPs confirmed that the quality of the recorded signals was independent from the choice of measuring electrode. Therefore, performing the measurement series only with E2 was sufficient. The waveforms obtained from single in-vitro recordings are shown in Figure 8a. As there were no visible differences between the plots of the waveforms obtained with standard and adaptive mode, only the waveforms recorded in adaptive mode are shown. The beginning and end of each waveform were omitted as they were contaminated by filter onset-offset effects and did not contain any relevant information. The waveforms are also depicted without their offsets. The observed offset component varied randomly between measurements in a range of 0.5 ± 0.4 mV, which was supposedly caused by the auto-offset calibration mechanism of the instrumentation amplifier. Moreover, repeated experiments with the generator disconnected from the petri dish confirmed that the generator had no influence on the observed offset. Because of the random offset component, we limited the adjustable amplitude of the sinusoidal input signal to a maximum of 4 mV in order to guarantee that the whole signal (burst plus offset) was completely within the input signal range of the recording system. The waveforms also showed that there was a small transient signal with a slope of approximately 0.2 mV/ms superimposing on each sinusoidal burst. This transient was thought to be related to residual stimulation artifact as it did not appear in the absence of a preceding stimulation pulse.

**Figure 9:**
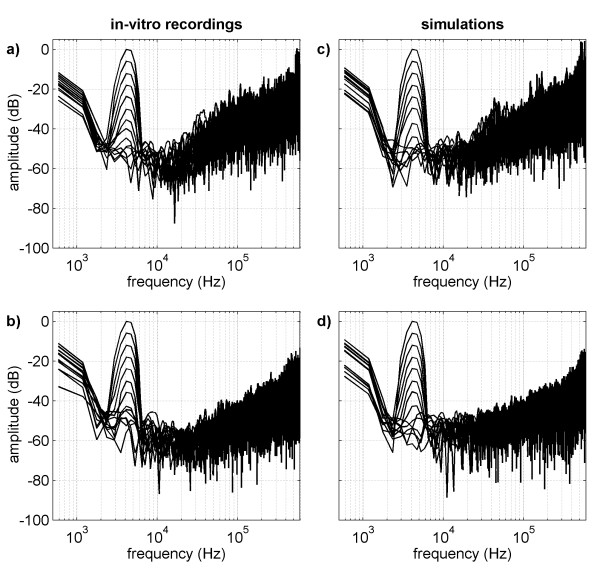
**Spectra of single SDM outputs for standard and adaptive mode**. The spectra of the in-vitro recordings in standard (a) and adaptive mode (b) are shown on the left side. On the right side the spectra of the corresponding simulations with standard (c) and adaptive mode (d) are depicted.

The spectra of the recordings obtained in standard and adaptive mode are depicted in Figure 9a and 9b respectively. Similar results were obtained in both modes in regard to the main signal component at the frequency of 4.3 kHz, and in regard to the low frequency components which are mainly due to the observed random offset. Each time the amplitude of the generator signal was reduced, the main signal component was decreased by 6 dB until noise level was reached.

The noise level comprised of two parts. For frequencies above approximately 20 kHz noise was dominated by quantization noise, while for low frequencies the noise level was dominated by amplifier noise, which is independent of the used SDM mode. While the high frequency components of the SDM quantization noise are damped effectively depending on the used low-pass filter, the amplifier noise within the signal band poses a limiting factor on the measurement of small signals and thus determines the resolution of the tested recording system.

### Averaged recordings with standard and adaptive mode

Compared to single in-vitro measurements the waveforms obtained by averaging 100 recordings showed a better resolution of small signals. This was true for recordings obtained in both SDM modes.

**Figure 10:**
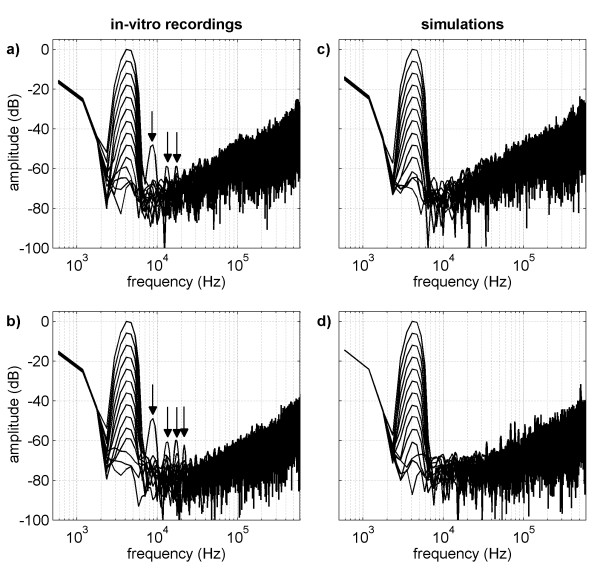
**Spectra of mean SDM outputs for standard and adaptive mode**. The spectra of the in-vitro recordings in standard (a) and adaptive mode (b) are shown on the left side. On the right side the corresponding spectra obtained from the simulations with standard (c) and adaptive mode (d) are depicted. Compared with the spectra in Figure 9 the noise level is reduced by 20 dB. The harmonic components are marked with arrows.

The reason for the improved resolution is revealed in the corresponding spectra shown in Figure 10a and 10b. The main signal components with small amplitudes are resolved because, compared to single measurements, averaging 100 recordings reduces the noise level by 20 dB. Due to the decrease in noise level the higher harmonic components are detectable. Although higher harmonic components are already included in the generator output signal (see Figure 7b), the harmonic component at approximately 9 kHz is too large to be ascribed to imperfections in the generator. In pin-board experiments with direct access to the pins of the ASIC we verified that for signals close to the limits of the input range of the recording system, higher harmonic components were generated by nonlinearities of the instrumentation amplifier.

### System linearity and resolution

**Figure 11:**
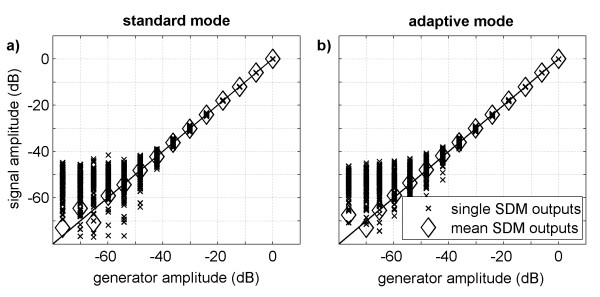
**Linearity of the recording system**. The main signal amplitudes of the in-vitro recordings in standard (a) and adaptive mode (b) are plotted against the corresponding generator amplitudes. Experimental data of 100 single SDM outputs (x-marks) and data obtained from the mean SDM outputs (diamonds) are depicted for each generator amplitude. As a reference, the behavior of an ideal linear system is included in the plots (solid line). For detectable signals recording is linear in both modes.

Figure 11 illustrates the relationship between the amplitude of the measured signal burst and the output amplitude of the generator. The plots indicate that the system shows linear behavior until signal amplitudes approach the noise level. Thus, for single measurements with standard and adaptive mode the recording system is expected to behave linearly down to about -40 dB. Averaging 100 recordings reduces the noise level by 20 dB in relation to the noise level of single recordings. With this lower noise level, the linear behavior of the recording system is revealed down to approximately -60 dB for standard mode and to approximately -65 dB for adaptive mode.

In this study the resolution of the EAP recording system was based on the reproducibility of the recorded waveforms. To achieve this, it is necessary to have stable and reproducible test signals, which in these experiments were delivered by the signal generator. With in-vitro measurements a resolution of about 80 μV was determined for single recordings, and a resolution of about 10 μV was determined when using the average of 100 recordings. The increase in resolution can be explained by the lower noise level observed with averaged recordings rather than with single recordings. The determined resolutions were almost independent from signal amplitude and SDM mode. It should be noted that generally the particular value of the resolution depends on the pass-band width of the used low-pass filter, which determines the actual noise power within the waveforms. The in-vitro experiments in this study were performed with sinusoidal test signals. The relationship between resolution and signal type needs to be further investigated. The resolution determined in our experiments is of the same magnitude as the resolution limit found in recordings with a MED-EL SONATA_TI_^100 ^user [22]. Bahmer et al. showed amplitude growth sequences where EAP amplitudes were resolved down to approximately 20 μV. In their experiments they used an averaging of 50 recordings and a low-pass filter with a drop-off of 6 kHz -3 dB and 16 kHz -60 dB.

### Simulations

When comparing the waveforms obtained from the simulations of each mode, no relevant differences were found in between them. Therefore, only the waveforms obtained in adaptive SDM are plotted in Figure 8b. The waveforms are depicted without offset and without the regions affected by filter onset-offset effects. The simulation results agree with the experimental results except for the presence of the transient signal due to residual stimulation artifact, as this was not considered in the mathematical model.

For a better comparison, the spectra of the simulations were plotted against the corresponding in-vitro results. The spectra obtained with both SDM modes are shown in Figure 9c and 9d for single recordings, and in Figure 10c and 10d for averaged recordings. The noise level and noise shape observed in the simulations, as well as the amplitude of the main signal and the offset components, are consistent with the experimental results. Higher harmonic components emerging in the in-vitro recordings are missing in the spectra of the simulations as the mathematical model included a linear amplifier.

## Conclusions

The sigma-delta based EAP recording system of the MED-EL PULSARCI^100 ^cochlear implant operates reliably in both, standard and adaptive mode.

Determination of system linearity and system resolution is limited by the observed noise level.

When using averaged recordings (100 measurements) the system revealed linear behavior down to about -60 dB. The corresponding system resolution was 10 μV for sinusoidal signals, and was almost independent of SDM mode and signal amplitude.

The good agreement between the simulations and the EAP recording results justifies the use of the mathematical model for describing the tested EAP recording system.

## Competing interests

PS is a staff member of Research and Development at MED-EL GmbH.

## Authors' contributions

CZ and PS developed the basic concept of the in-vitro experiments. CN and MZ carried out the in-vitro recordings and analyzed the data. The software code for the simulations of the standard and adaptive SDMs was provided by CZ and PS. CN wrote the draft of the publication, which was discussed, criticized, and partly rewritten to clarify the presentation together with CZ, PS, and MZ. CZ was responsible for the EAP-signal processing concept and for implementing the system. CZ also advised on technical concerns. All authors read and approved the final manuscript.
